# The Psychological Burden of the 2023 Sudanese Conflict: PTSD Prevalence and Coping Mechanisms

**DOI:** 10.1192/bjo.2025.10146

**Published:** 2025-06-20

**Authors:** Walaa E. A. Abdallah, Weam M. M. Ahmed, Mujtaba Mohamed, Abdulhadi M. A. Mahgoub, Danya Ibrahim

**Affiliations:** ^1^Faculty of Medicine, International University of Africa, Khartoum, Sudan; ^2^Khartoum University, Faculty of Medicine, Khartoum, Sudan; ^3^Faculty of Medicine, University of Gezira, Madani, Sudan

## Abstract

**Aims:** This study aimed to identify the prevalence of post-traumatic stress disorder (PTSD) and to assess coping strategies among Sudanese individuals. Also, to evaluate the relationship between PTSD and coping mechanisms with sociodemographic characteristics.

**Methods:** This study utilized a cross-sectional design to assess PTSD and coping strategies in 716 Sudanese adults affected by war, selected through convenience sampling. Participants completed a Google Form questionnaire that included sociodemographic data, the PTSD Checklist for DSM–5 (PCL-5), and a coping scale. The analysis was conducted using SPSS software version 26, applying various statistical tests to evaluate relationships and differences.

**Results:** The findings revealed that nearly 43% of the sample met the criteria for a potential diagnosis of PTSD. About 69% of participants were female, with a median age of 23 years. Most participants were single (81%) and had been externally displaced (51%). Coping strategies varied among the participants: 34% focused on improving their habits, while others used reflective approaches (36%), sought positive perspectives (30%), employed humour (21%), or chose to wait for problems to resolve on their own (17%). Family income was significantly associated with PTSD symptoms (p=0.020). Participants with higher PTSD symptoms exhibited lower coping effectiveness, and both age and marital status significantly influenced coping mechanisms (p=0.027 and 0.037, respectively). Those who had been living in conflict zones for over six months reported the highest coping scores.

**Conclusion:** Our findings confirmed a high level of PTSD symptoms among the participants. Duration of residency in the conflict zone impacted the coping adopted and income played a crucial role in developing PTSD. This study underscores the need for urgent specific psychological support for individuals affected by the conflict. Further research is required to foster mental well-being, and put more attention to this issue.

